# Fractal dimension: analyzing its potential as a neuroimaging biomarker for brain tumor diagnosis using machine learning

**DOI:** 10.3389/fphys.2023.1201617

**Published:** 2023-07-17

**Authors:** Dheerendranath Battalapalli, Sreejith Vidyadharan, B. V. V. S. N. Prabhakar Rao, P. Yogeeswari, C. Kesavadas, Venkateswaran Rajagopalan

**Affiliations:** ^1^ Department of Electrical and Electronics Engineering, Birla Institute of Technology and Science Pilani, Hyderabad Campus, Hyderabad, India; ^2^ Department of Pharmacy, Birla Institute of Technology and Science Pilani, Hyderabad Campus, Hyderabad, India; ^3^ Department of Imaging Sciences and Interventional Radiology, Sree Chitra Tirunal Institute for Medical Sciences and Technology, Trivandrum, India

**Keywords:** brain magnetic resonance imaging, texture feature, fractal dimension, biomarker, low-grade glioma, high-grade glioma

## Abstract

**Purpose:** The main purpose of this study was to comprehensively investigate the potential of fractal dimension (FD) measures in discriminating brain gliomas into low-grade glioma (LGG) and high-grade glioma (HGG) by examining tumor constituents and non-tumorous gray matter (GM) and white matter (WM) regions.

**Methods:** Retrospective magnetic resonance imaging (MRI) data of 42 glioma patients (LGG, n = 27 and HGG, n = 15) were used in this study. Using MRI, we calculated different FD measures based on the general structure, boundary, and skeleton aspects of the tumorous and non-tumorous brain GM and WM regions. Texture features, namely, angular second moment, contrast, inverse difference moment, correlation, and entropy, were also measured in the tumorous and non-tumorous regions. The efficacy of FD features was assessed by comparing them with texture features. Statistical inference and machine learning approaches were used on the aforementioned measures to distinguish LGG and HGG patients.

**Results:** FD measures from tumorous and non-tumorous regions were able to distinguish LGG and HGG patients. Among the 15 different FD measures, the general structure FD values of enhanced tumor regions yielded high accuracy (93%), sensitivity (97%), specificity (98%), and area under the receiver operating characteristic curve (AUC) score (98%). Non-tumorous GM skeleton FD values also yielded good accuracy (83.3%), sensitivity (100%), specificity (60%), and AUC score (80%) in classifying the tumor grades. These measures were also found to be significantly (*p* < 0.05) different between LGG and HGG patients. On the other hand, among the 25 texture features, enhanced tumor region features, namely, contrast, correlation, and entropy, revealed significant differences between LGG and HGG. In machine learning, the enhanced tumor region texture features yielded high accuracy, sensitivity, specificity, and AUC score.

**Conclusion:** A comparison between texture and FD features revealed that FD analysis on different aspects of the tumorous and non-tumorous components not only distinguished LGG and HGG patients with high statistical significance and classification accuracy but also provided better insights into glioma grade classification. Therefore, FD features can serve as potential neuroimaging biomarkers for glioma.

## Highlights

⁃ A comprehensive fractal dimension (FD) feature set was extracted from tumorous and non-tumorous brain regions.
⁃ Texture features were also measured in the tumorous and non-tumorous brain regions.
⁃ FD features showed superior performance in distinguishing low-grade glioma (LGG) from high-grade glioma (HGG).
⁃ Interestingly, non-tumorous FD features also distinguished LGG from HGG, suggesting that the pathophysiological process of the tumor may not be local.

## 1 Introduction

Brain tumors are defined as the abnormal and uncontrolled growth of brain cells (McFaline-Figueroa and Lee, 2018). As a consequence, it affects the metabolic and functional activities of the brain. Brain tumors are categorized into different types based on their origin from the brain cells. Glioma is the most frequent type of primary brain tumor that originates from the brain glial cells (Li et al., 2018; Ostrom et al., 2019). Glioma is typically categorized into low-grade glioma (LGG) and high-grade glioma (HGG). HGG accounts for 75%–80% of the primary brain tumors (Louis et al., 2016). They are usually cancerous tumors whose structure appears in an amorphous/irregular form in radiological images compared to well-circumscribed LGG tumors. At least three-fifths of the adults are diagnosed with HGG, which has an aggressive growth rate, leading to mortality within 2 years after diagnosis (Chinot et al., 2014; Gilbert et al., 2014). Moreover, brain tumors are prevalent and catastrophic in children (Smith and Reaman, 2015). Furthermore, pediatric brain tumor survivors experience long-term adverse effects due to treatment procedures in their later adulthood, which is an alarming concern (Brinkman et al., 2016; Chemaitilly et al., 2016).

Identifying the tumor grade is important for choosing appropriate treatment strategies, procedures, and prognostic evaluation. Clinically, tumor grading is performed by collecting histopathological (Pollack et al., 2019; Figarella-Branger et al., 2022) samples using biopsy. However, there are certain circumstances where a collection of tissue samples from the wrong brain region or inadequate sample collection leads to misinterpretation of the tumor grade (Sugahara et al., 1999). These drawbacks can be overcome if neuroimaging could aid in accurate tumor diagnosis and grading. Among neuroimaging modalities, magnetic resonance imaging (MRI) is radiation-free and can provide high contrast to visualize brain soft tissues (Arslan et al., 2016). Conventional MRI sequences such as T1-weighted, fluid-attenuated inversion recovery (FLAIR), and T2-weighted and T1-weighted contrast-enhanced images are routinely used to qualitatively demarcate tumor regions (edema, enhanced tumor region, and whole tumor regions) and their shape (Donahue et al., 2008; Zacharaki et al., 2009; Li et al., 2018). Note, in this manuscript, we refer to the region of enhancement, a common feature of malignancy observed in the MRI of HGG tumor patients, as an enhanced tumor region. Nevertheless, such qualitative evaluation of MRI is prone to errors in distinguishing tumors from non-tumoral lesions, such as ischemia, and in tumor grading. Even with the use of advanced MRI techniques, such as diffusion tensor imaging, it is often difficult to differentiate the tumor grade (Sinha et al., 2002). *In vivo* tumor studies show that malignant tumor cells invade and develop branching, which makes the tumor structure amorphous/irregular. In addition, this cell invasion and branching affect the morphometry of the non-tumorous brain regions (Deisboeck et al., 2001; Iftekharuddin et al., 2003). These microscopic changes in due course will affect the tissue morphometry, which can be detected using conventional T1-weighted MRI sequences. Detecting these shape morphometric changes non-invasively using quantitative MRI measures may help in identifying neuroimaging-based biomarkers which can overcome the challenges of biopsy and qualitative tumor diagnosis. The quantitative measures of shape morphometry, therefore, may aid in the differentiation, diagnosis, and treatment of tumors.

Previous studies (Zacharaki et al., 2009; Skogen et al., 2016; Zhang et al., 2017; Suárez-García et al., 2020) used texture-based shape morphometric features such as angular second moment, contrast, inverse difference moment, correlation, and entropy from the gray-level co-occurrence matrix. Some other studies (Durmo et al., 2018), instead of shape features, focused on volumetric features of the tumor and its constituents derived from conventional MRI sequences like T1-weighted, T2-weighted, T1-weighted contrast-enhanced, and FLAIR images. All the aforementioned studies primarily focused on the tumor and its constituent regions (whole tumor, enhanced tumor, and edema regions) to differentiate LGG and HGG. We know that the aforementioned texture and volumetric features do not capture the shape morphometry characteristics such as the change in the boundary caused by the irregular growth pattern and branching of the tumor into the surrounding tissues. Fractal dimension (FD) is a quantitative shape morphometry approach that is widely used in detecting abnormal changes in the tissue boundary caused by neurological disorders. FD was used to analyze shape complexity changes in neurological disorders such as multiple sclerosis, Alzheimer’s disease, and traumatic brain injury (Zhang et al., 2008; Smitha et al., 2015). FD analysis was also employed in brain tumor studies (Zhang et al., 2007; Smitha et al., 2015). FD was found to be effective in examining the complexities in the tissue structure brought out by brain tumors (Zhang et al., 2016; Maipas et al., 2018). In general, the FD values tend to increase as the shape of the object becomes more irregular (Smitha et al., 2015). Since the tumor growth pattern varies between LGG (well-circumscribed) and HGG (irregular) tumors, FD analysis can play a vital role in differentiating LGG from HGG. Previous FD studies in brain tumors used small sample-sized datasets (Tang and Nan Wang, 2005; Rose et al., 2009; Zhang et al., 2016; Maipas et al., 2018). In addition, to the best of our knowledge, FD analysis was performed only for the whole tumor boundary structure to distinguish tumor grades. Therefore, an exploratory/comprehensive FD analysis of not only the whole tumor but also its constituents is needed.

Therefore, in this study, we aimed to comprehensively investigate a) the potential of FD as a neuroimaging biomarker obtained from the tumor constituents and also from non-tumorous gray matter (GM) and white matter (WM) brain regions. It is noteworthy that even though brain tumor is a local phenomenon, modeling studies have shown that cell invasion and branching affect non-tumorous brain regions; b) different aspects of FD, namely, fractal of the tumor’s general structure, tumor boundary fractal, and fractal of the tumor skeleton, and similarly, the fractal of the general structure, boundary, and skeleton of non-tumorous GM and WM brain regions; and c) efficacy of FD features compared to commonly used texture features. To the best of our knowledge, no other study has assessed the efficacy of the aforementioned three different aspects (i.e., general structure, boundary, and skeleton) of FD measures in distinguishing LGG from HGG. Therefore, FD analysis can be used as a promising radiological biomarker for the diagnosis/classification of LGG and HGG tumors. Furthermore, FD analysis of non-tumorous GM and WM regions in LGG and HGG patients may shed light on the non-local phenomenon of brain tumors.

We hypothesize that a) FD analysis of different aspects (general structure, boundary, and skeleton) of the tumor constituents can provide better insights into the shape morphometry changes brought out by the pathophysiological processes between LGG and HGG. These different FD measures can also achieve better accuracy, sensitivity, specificity, and area under the receiver operating characteristic curve (AUC) score. To verify this hypothesis, a comparison was performed between the texture features (angular second moment, contrast, inverse difference moment, correlation, and entropy) measured in the tumorous and non-tumorous brain regions of LGG and HGG patients.

## 2 Materials and methods

### 2.1 Data acquisition

For this study, the data of 42 glioma patients LGG, n = 27 and HGG, n = 15 were acquired from Sree Chitra Tirunal Institute of Medical Science and Technology, Thiruvananthapuram. The Internal Ethics Committee at the hospital approved this study, and the study was conducted in accordance with the Declaration of Helsinki. The Institutional Ethics committee (IEC Regn No. ECR/189/Inst/KL/2013/RR-16) at Sree Chitra Tirunal Institute of Medical Science and Technology, Thiruvananthapuram, India, approved this study, waiving patient informed consent as this is a retrospective study. The approval number is IEC/1177. All procedures were performed under relevant guidelines.

### 2.2 Imaging protocol

All the patients underwent scanning using a 1.5T Siemens MRI scanner (MAGNETOM Avanto, Erlangen, Germany). The MRI sequences include 1) 2D T2-weighted images with slice thickness = 5 mm, in-plane resolution = 512 × 448, repetition time (TR) = 5,860 ms, and time of echo (TE) = 110 ms; 2) FLAIR images were acquired with in-plane resolution = 512 × 448, slice thickness = 5 mm, TR = 9,000 ms, inversion time (TI) = 2,500 ms, and TE = 89 ms; 3) 2D T1-weighted images were acquired with slice thickness = 5 mm, in-plane resolution = 320 × 270, TR = 468 ms, and TE = 11 ms; and 4) 3D gradient echo was used to acquire T1-weighted contrast-enhanced images whose imaging parameters include slice thickness = 0.9 mm, in-plane resolution = 512 × 464, TR = 9 ms, and TE = 3.34 ms.

### 2.3 Mathematical details of fractal dimension analysis

Mathematically, an object is said to be a fractal if it possesses self-similarity, fine details and lacks Euclidean dimension (Mandelbrot and Wheeler, 1983; Lopes and Betrouni, 2009). Fractal objects obey the power law relationship, given in Eq. 1 (Mandelbrot and Wheeler, 1983):

$$N=S^{-D}.$$ (1)

In the aforementioned equation, D is the dimension to be estimated for an object/structure based on the given scale S by evaluating the self-similar units called N. The aforementioned power law relationship is non-linear. It is converted into a linear expression by taking logarithms on both sides, as shown in Eq. 2:

$$D=\log_{10}N/\log_{10}\left(1/S\right),$$ (2)

where D is the fractal dimension. In a realistic practical scenario, the exact scale value S and the fractal dimension D value in Eq. 2 are unknown. Usually, the scale value S is varied over a range of values, and the corresponding number of self-similar units N for each scale is measured. Using the measured number of units N and the chosen scale value S for the considered range of S values, a linear regression fit is performed to estimate the fractal dimension D value from Eq. 2. The number of units covering the object for a given scale is obtained by using a popular method called the box-counting (BC) method (Ashraf et al., 2021). Briefly, in the BC method, the pattern/image is covered by a grid of boxes whose size depends on the corresponding scale S. The N in Eq. 2 represents the number of boxes that covers the object/pattern/image for a given scale S.

Briefly, the FD estimation steps include the box-counting method applied to our tumorous and non-tumorous binary images. We incrementally placed boxes of different sizes (S) on the binary tumor region of general structure, boundary, and skeleton images to count the number of boxes (N) it takes to fully cover the structure to calculate the FD values. Then, a regression line with the best fit was plotted between the log-transformed N and S to estimate the FD values using Eq. 2.

### 2.4 Data processing

**FIGURE 1:**
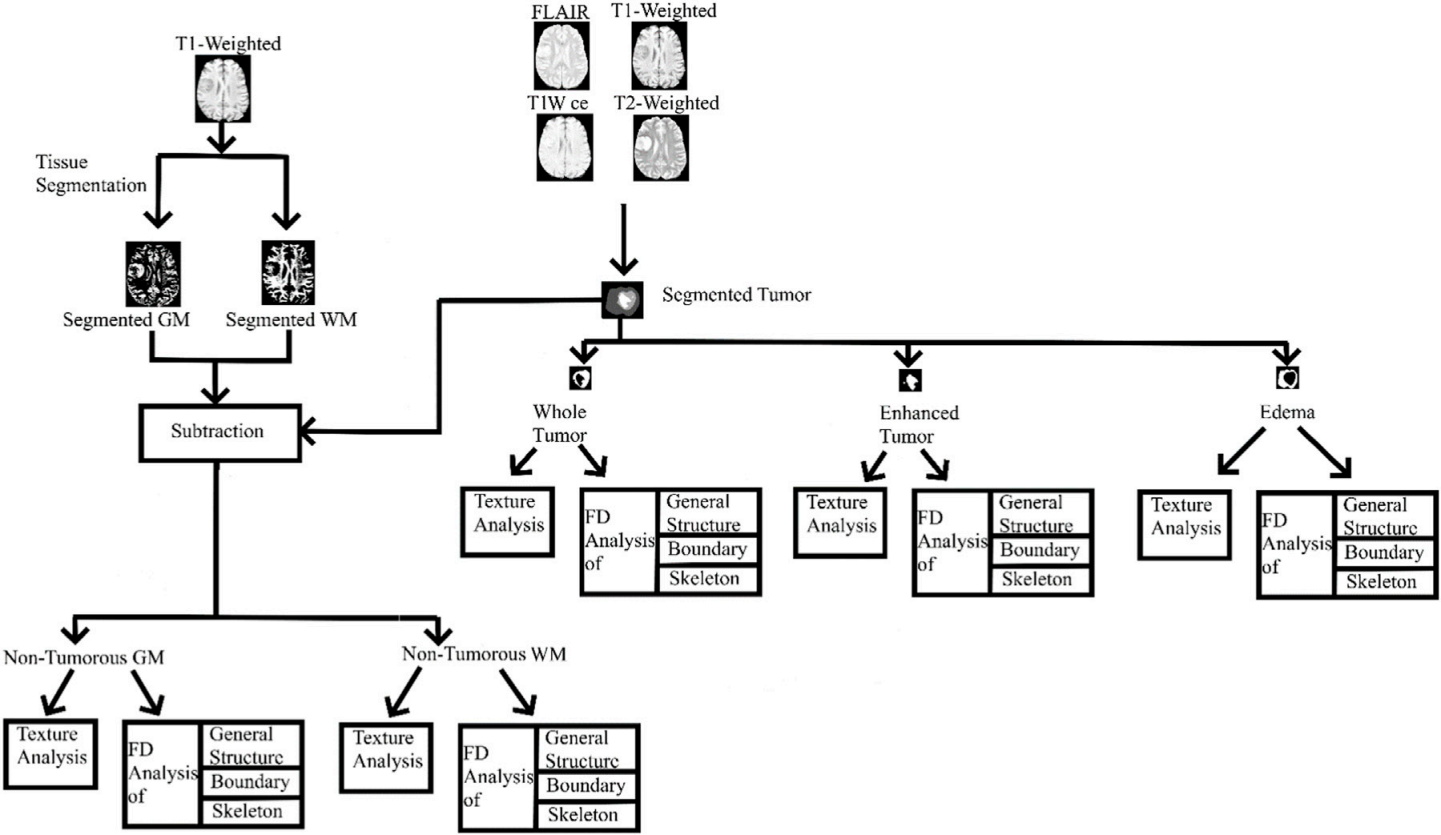
Entire workflow. GM, gray matter; WM, white matter; T1W ce, T1-weighted contrast-enhanced image; FD, fractal dimension.

Image pre-processing steps include: a) brain extraction was performed using the FSL BET tool (version 6.0.4, https://fsl.fmrib.ox.ac.uk/fsl/fslwiki/FslInstallation) with a fractional intensity threshold = 0.5 and a vertical gradient in the fractional intensity threshold = 0; b) brain extracted images were then corrected for intensity inhomogeneity using the default settings of the FSL FAST tool. The tumor regions from the MRI image sequences were segmented using the ITK-SNAP tool (http://www.itksnap.org/pmwiki/pmwiki.php). Tumor segmentation was performed under the supervision of an experienced board-certified radiologist (one of the authors); c) Statistical Parametric Mapping (https://www.fil.ion.ucl.ac.uk/spm/software/spm12/) software was used with default settings to segment the GM and WM regions based on a unified segmentation algorithm which uses prior tissue probability maps to further process and obtain the non-tumorous regions for FD analysis; d) these pre-processed images were subjected to texture analysis and FD analysis. The workflow is shown in Figure 1.

#### 2.4.1 Texture analysis

Gray-level co-occurrence matrix-based texture analysis was performed on the tumorous and non-tumorous brain regions using ImageJ software (Java 8 version, https://imagej.nih.gov/ij/download.html) using the Texture Analyzer plugin (https://imagej.nih.gov/ij/plugins/texture.html). Initially, the images were converted to 8-bit grayscale images, and then the default settings with square regions of interest were used. These images were then processed in batches using a custom-written macros program and an Excel Writer plugin for transferring the numerical features to Excel format (https://imagej.nih.gov/ij/plugins/excel-writer.html). Features including angular second moment, contrast, inverse difference moment, correlation, and entropy were calculated for whole tumor, enhanced tumor, edema, non-tumorous GM, and WM. A total of 25 features (five aforementioned texture features are calculated for each of the tumorous and non-tumorous regions) were extracted for each LGG and HGG patient.

#### 2.4.2 Fractal dimension analysis of tumor and its constituents in LGG and HGG patients

A custom MATLAB code was written *in situ* for fractal analysis using the box-counting function (https://in.mathworks.com/matlabcentral/fileexchange/13063-boxcount). FD analysis was performed on the tumor regions (i.e., whole tumor, enhanced tumor, and edema region) which were segmented from the MRI of LGG and HGG patients using the ITK SNAP tool (version 3.8, http://www.itksnap.org/pmwiki/pmwiki.php). The aforementioned segmented tumor regions were then binarized and subjected to FD calculation of their general structure, boundary, and skeleton.

**FIGURE 2:**
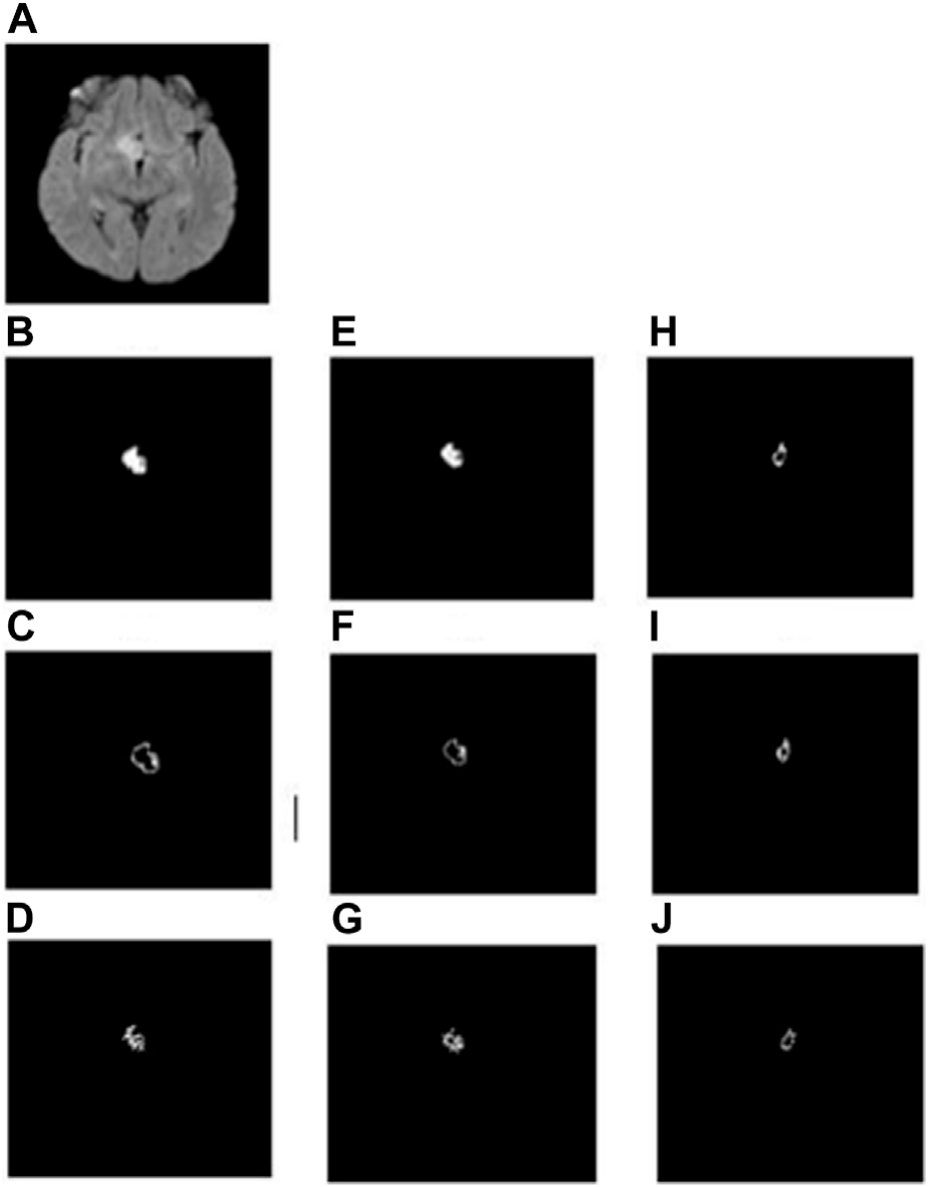
**(A)** FLAIR image of a typical LGG patient, **(B–D)** general structure, boundary, and skeleton of the whole tumor region, **(E–G)** general structure, boundary, and skeleton of the edema region, and **(H–J)** general structure, boundary, and skeleton of the enhanced tumor region, respectively.

**FIGURE 3:**
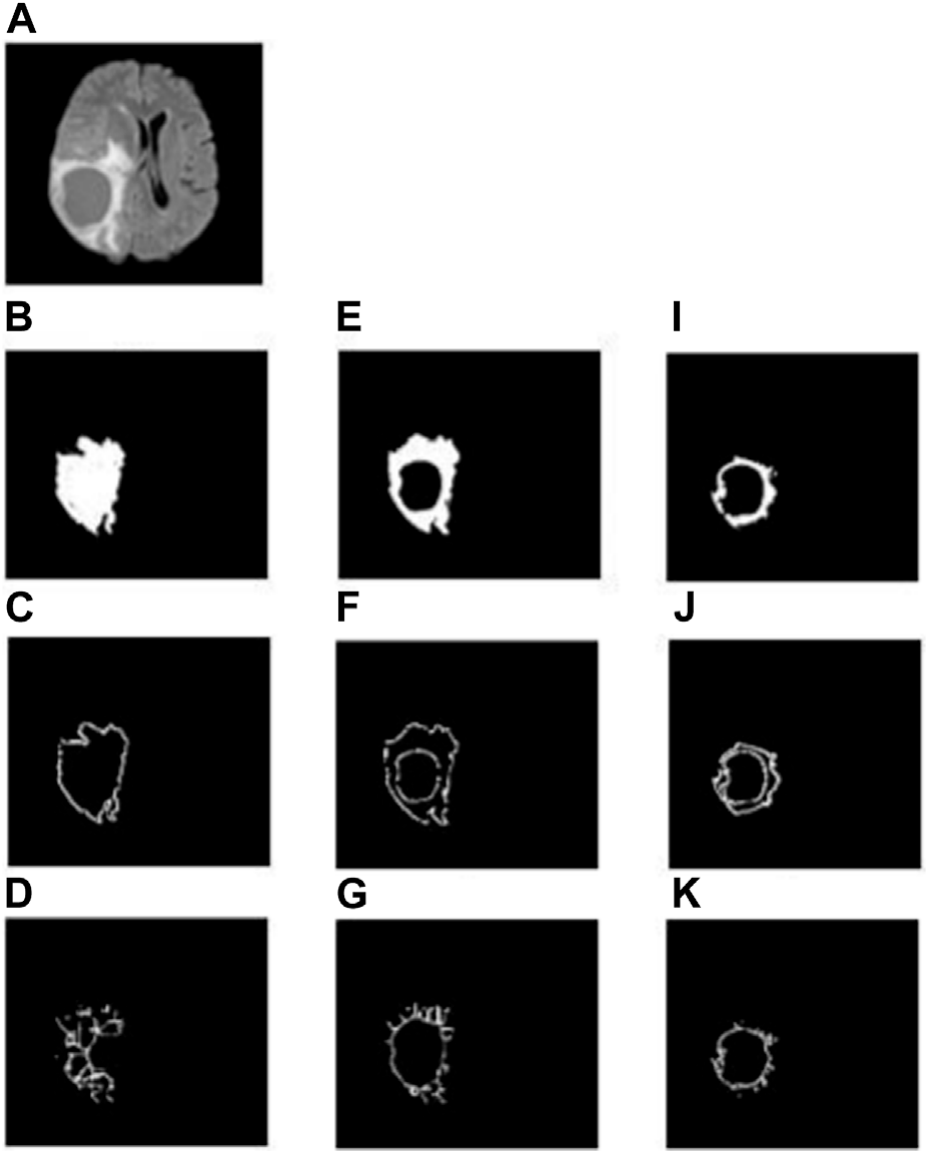
**(A)** FLAIR image of a typical HGG patient, **(B–D)** general structure, boundary, and skeleton of the whole tumor region, **(E–G)** general structure, boundary, and skeleton of the edema region, **(H–J)** general structure, boundary, and skeleton of the enhanced tumor region, respectively.

In the whole tumor general structure FD analysis, the boxes which covered the entire tumor region were considered while estimating the FD values. For the boundary FD structure analysis, the voxels within the tumor regions were removed by leaving out only the boundary voxels. This was performed by using the MATLAB morphological operation function “bwmorph.” Then, FD values for the boundary structure were estimated by counting the number of boxes that cover the boundary voxels. Similarly, in the case of skeleton FD analysis, the boundary voxels of the tumor region were removed without changing their general structure. This involves shrinking the input image until the region of interest is 1 pixel wide and equidistant from the image boundaries. The morphological operation was accomplished using “bwmorph,” a built-in MATLAB function. Finally, the FD value was calculated for the skeleton by counting the number of boxes required to cover the skeleton of the tumor region. Figures 2, 3 show the general structure, boundary, and skeleton of a typical LGG and HGG patient. In total, we have nine FD features (three for the whole tumor, three for enhanced tumor, and three for edema, where the three features are general structure, boundary, and skeleton) of the aforementioned tumorous regions.

#### 2.4.3 Fractal dimension analysis of non-tumorous regions in LGG and HGG patients

**FIGURE 4:**
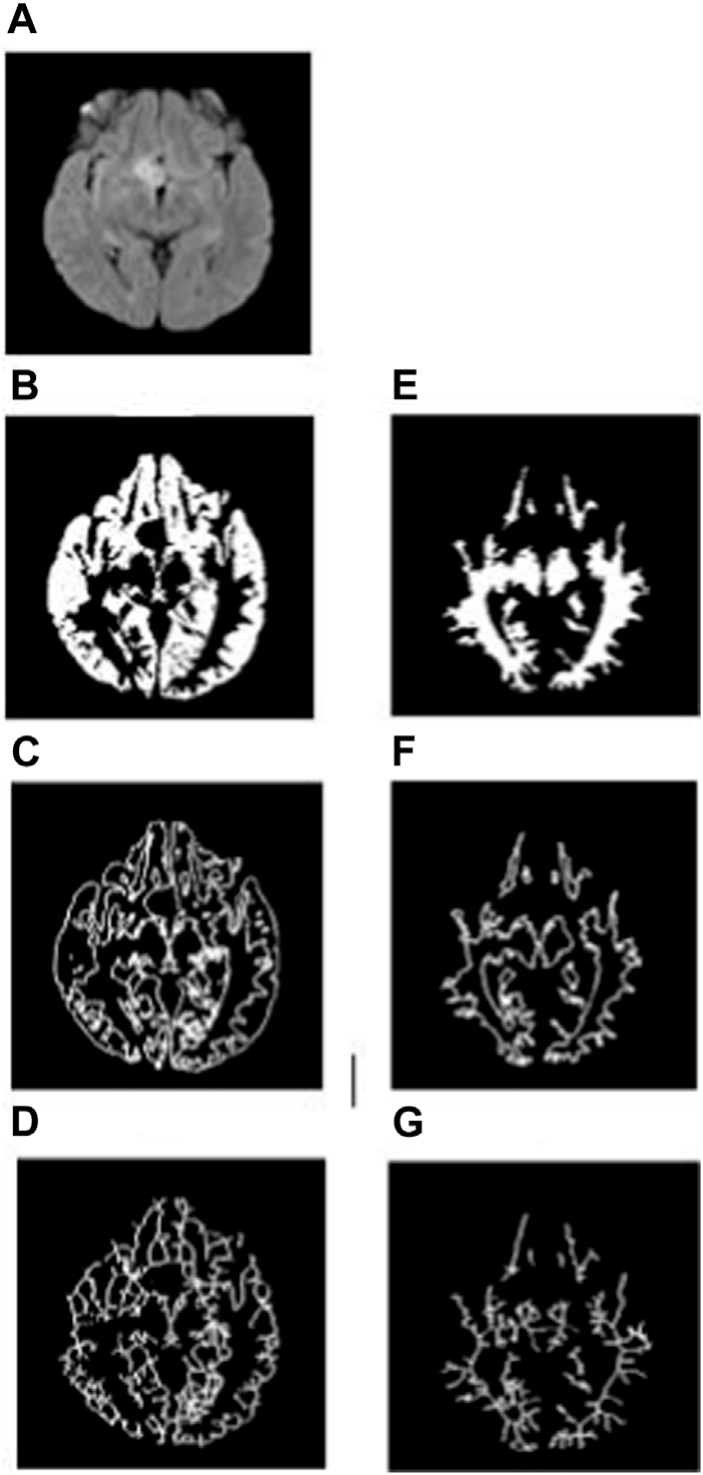
**(A)** FLAIR image of a typical LGG patient, **(B–D)** general structure, boundary, and skeleton of the non-tumorous GM region, and **(E–G)** general structure, boundary, and skeleton of the non-tumorous WM region.

**FIGURE 5:**
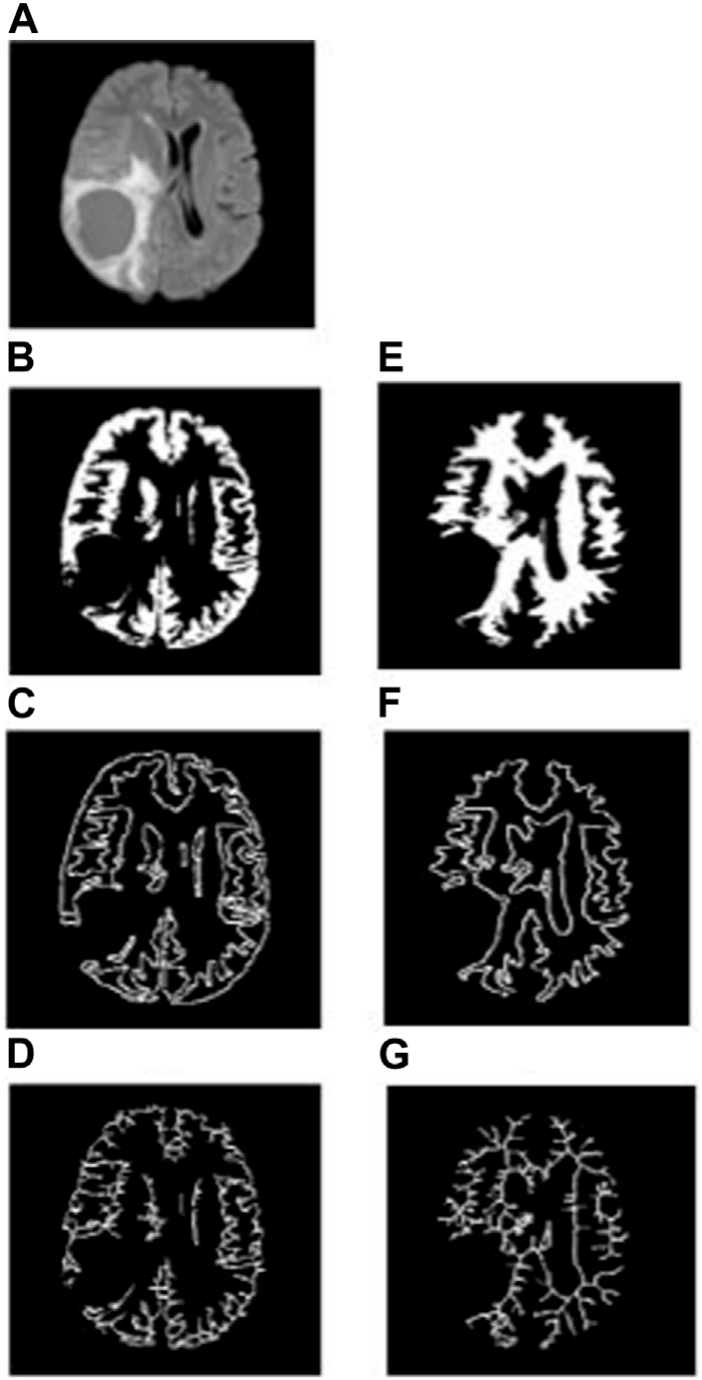
**(A)** FLAIR image of a typical HGG patient, **(B–D)** general structure, boundary, and skeleton of the non-tumorous GM region, and **(E–G)** general structure, boundary, and skeleton of the non-tumorous WM region.

To analyze the non-tumorous regions, first, we segmented the GM and WM from the T1-weighted MRI using SPM12 software. Furthermore, the corresponding tumor region was subtracted from the GM and WM regions to ensure that only the non-tumorous brain regions remain, as shown in Figure 1. Then, on the non-tumorous whole brain, GM, and WM images, FD values for the general structure, boundary structure, and skeleton were obtained using the box-counting method by adopting similar steps as given in the previous section. Figures 4, 5 show the general structure, boundary, and skeleton of a typical LGG and HGG patient. In total, we have six FD features for the aforementioned non-tumorous regions (three for GM and three for WM, where the three features are general structure, boundary, and skeleton).

### 2.5 Statistical inference and machine learning

The machine learning codes were written in Python version 3.9.7 using NumPy, Matplotlib, and sklearn libraries in the Jupyter Notebook. All machine learning algorithms were implemented for texture and FD features separately to classify LGG and HGG tumors. The following steps were performed for both the texture and FD features. For each patient, we measured 25 texture features and 15 FD features. Since the dataset is unbalanced (27 LGG and 15 HGG patients) and to avoid underfitting/overfitting, we used five-fold cross-validation. We performed five-fold cross-validation with 70% of the data for training and 30% for testing. Support vector machine (SVM) algorithms with linear, polynomial, and radial basis function (RBF) kernels were considered. The kernel that yielded the best evaluation metric results was selected and reported in this study. We then performed receiver operating characteristic (ROC) analysis to obtain an AUC score for the general structure, boundary, and skeleton region FD values for the tumor constituents and non-tumorous regions. We then compared the AUC score of all the measurements to assess the discriminative power of FD analysis in classifying LGG from HGG tumors.

Statistical inference tests were performed separately for the texture and FD features to distinguish LGG and HGG patients. Student’s t-test or the Mann–Whitney *U* test was used based on the data meeting the assumptions of normality. A two-sided significance level of *p* < 0.05 was used after correcting for multiple comparisons using a false discovery rate. Statistical comparisons were performed using MedCalc open ware software (Windows version 20.022, https://www.medcalc.org/download/) and IBM SPSS Statistics software (version 25, https://www.ibm.com/products/spss-statistics).

## 3 Results

### 3.1 Texture analysis statistical inference and machine learning results

For enhanced tumor regions, the contrast, correlation, and entropy features were found to be statistically different between LGG and HGG patients (*p* = 0.00001). Contrast and entropy values were found to be higher in HGG patients (contrast = 162.75 ± 103.14 (mean ± standard deviation), entropy = 0.08 ± 0.04) than LGG patients (contrast = 13.53 ± 7.51 (mean ± standard deviation); entropy = 0.006 ± 0.004). Furthermore, the correlation measure was found to be reduced in HGG (0.006 ± 0.005) patients compared to LGG patients (0.03 ± 0.02).

In edema regions, inverse difference moment (IDM) and contrast values (*p* = 0.0001) and angular second moment (ASM) and entropy values (*p* = 0.01) were found to be significantly different between LGG and HGG patients. IDM was found to be greater in HGG patients (0.99 ± 0.0007) than in LGG patients (0.98 ± 0.0006), whereas the contrast measure was found to be reduced in HGG patients (95.59 ± 42.04) compared to LGG patients (172.68 ± 48.61). Similarly, ASM values were found to be lower in LGG patients (0.95 ± 0.01) than in HGG patients (0.97 ± 0.01), and entropy values were found to be higher in LGG patients (0.106 ± 0.023) than in HGG patients (0.069 ± 0.029).

Considering the whole tumor region, contrast and IDM features (*p* = 0.03) revealed significant differences between LGG and HGG patients. Contrast measure was found to be lower in LGG patients (55.18 ± 40.28) than in HGG patients (60.942 ± 54.03), whereas IDM was found to be greater in LGG patients (0.998 ± 0.001) than in HGG patients (0.998 ± 0.001). No significant difference was observed when considering the texture features from non-tumorous regions. These results are given in Table 1.

**TABLE 1:** Statistical inference results (only significant results are shown, i.e., for *p*-value < 0.05) of the texture features from the tumorous regions. ASM, angular second moment; IDM, inverse difference moment.

Region	LGG (M ± SD)	HGG (M ± SD)	*p*-value
*Whole tumor*			
Contrast	55.18 ± 40.28	60.942 ± 54.032	0.01
IDM	0.99 ± 0.0074	0.998 ± 0.001	0.03
*Enhanced tumor*			
Contrast	13.53 ± 7.51	162.75 ± 103.14	0.00001
Correlation	0.03 ± 0.02	0.006 ± 0.005	0.00001
Entropy	0.006 ± 0.004	0.08 ± 0.04	0.00001
*Edema*			
IDM	0.98 ± 0.0006	0.99 ± 0.0007	0.0001
Contrast	172.68 ± 48.61	95.59 ± 42.04	0.0001
ASM	0.95 ± 0.01	0.97 ± 0.01	0.001
Entropy	0.106 ± 0.023	0.069 ± 0.029	0.001

M, mean; SD, standard deviation; *p < 0.05 (false discovery rate).

Among the three different kernels used for SVM, the linear kernel had superior performance (89% accuracy, 95% sensitivity, 90% specificity, and 90% AUC score) when trained with texture features of the enhanced tumor region. For all other texture features from tumorous and non-tumorous regions, the model accuracy, sensitivity, specificity, and AUC score were below 70%. These results are given in Table 2.

**TABLE 2:** SVM results for tumorous and non-tumorous brain regions when considering all the five texture features for each of the regions.

Region	Accuracy	Sensitivity	Specificity	AUC score
Whole tumor	60	57	60	56
Enhanced tumor	89	95	90	90
Edema	70	72	70	70
Non-tumorous GM	50	33	50	40
Non-tumorous WM	60	36	61	50

### 3.2 Fractal dimension analysis of the tumor and its constituents

FD general structure, boundary, and skeleton values measured from the whole tumor, enhanced tumor, and edema regions (i.e., in total, nine FD measures) were compared between LGG and HGG patients. Figures 2, 3 show the different structures of the tumor and its constituents on which FD estimates were performed in a typical LGG and HGG patient. Statistical analysis revealed that the FD skeleton of edema (*p* = 0.0009), FD skeleton of the enhanced tumor region (*p* < 0.0001), FD boundary of the whole tumor (*p* = 0.0105), and FD general structure of the enhanced tumor region (*p* < 0.0001) were significantly different between LGG and HGG patients. The remaining FD measures did not show any statistical significance in distinguishing the tumor grades. These results, along with mean and standard deviation values of the FD measures, are given in Table 3. In general, FD values were higher in HGG patients than in LGG patients. This is probably due to the irregular tumor structure observed in the MRI of HGG patients.

**TABLE 3:** Fractal dimension values of the tumorous region.

Region	LGG (M ± SD)	HGG (M ± SD)	*p*-value
*Skeleton*			
Whole tumor	0.901 ± 0.029	0.911 ± 0.022	0.2993
Edema	0.895 ± 0.020	0.929 ± 0.035	0.0009*
Enhanced tumor	0.802 ± 0.066	0.898 ± 0.062	<0.0001*
*Boundary*			
Whole tumor	0.923 ± 0.023	0.940 ± 0.021	0.0105*
Edema	0.941 ± 0.029	0.931 ± 0.022	0.3700
Enhanced tumor	0.932 ± 0.045	0.952 ± 0.048	0.2105
*General*			
Whole tumor	1.755 ± 0.087	1.767 ± 0.057	1.000
Edema	1.720 ± 0.100	1.744 ± 0.083	0.6043
Enhanced tumor	1.221 ± 0.132	1.628 ± 0.091	<0.0001*

M, mean; SD, standard deviation; *p < 0.05 (false discovery rate).

### 3.3 Fractal dimension analysis of non-tumorous brain regions

A similar kind of FD analysis was performed on the non-tumorous GM and WM structures. Table 4 shows the mean, standard deviation, and *p-*values for the general structure, boundary, and skeleton FD measures of non-tumorous GM and WM regions in LGG and HGG patients. Among the six different FD measures, the skeleton of GM (LGG = 0.942 ± 0.032 and HGG = 0.998 ± 0.024, *p* = 0.0001) and WM (LGG = 0.931 ± 0.035 and HGG = 0.955 ± 0.024, *p* = 0.0140) revealed a significant difference in their structural complexity between LGG and HGG patients.

**TABLE 4:** Fractal dimension values of the non-tumorous regions.

Region	LGG (M ± SD)	HGG (M ± SD)	*p*-value
*Skeleton*			
GM region	0.942 ± 0.032	0.998 ± 0.024	0.0001*
WM region	0.931 ± 0.035	0.955 ± 0.024	0.0140*
*Boundary*			
GM region	1.025 ± 0.045	1.075 ± 0.051	0.0025*
WM region	0.991 ± 0.056	1.012 ± 0.033	0.1610
*General*			
GM region	1.693 ± 0.063	1.662 ± 0.053	0.1296
WM region	1.699 ± 0.029	1.744 ± 0.072	0.0013*

M, mean; SD, standard deviation; *p < 0.05 (false discovery rate).

The WM boundary FD values failed to differentiate between LGG (0.991 ± 0.056 (mean ± standard deviation)) and HGG (1.012 ± 0.033), but the GM boundary (*p* = 0.0025) FD values revealed a significant difference between them (LGG = 1.025 ± 0.045 and HGG = 1.075 ± 0.051). In addition, the general structure of the WM region (*p* = 0.0013) FD values revealed a statistically significant difference between LGG (1.699 ± 0.029) and HGG (1.744 ± 0.072), while the general structure of the GM region remained statistically insignificant (LGG = 1.693 ± 0.063 and HGG = 1.662 ± 0.053). Future studies using diffusion tensor imaging and GM cortical thickness analysis may confirm our findings on significant differences in FD values between non-tumorous WM and GM regions. Figures 4, 5 show the general structure, boundary, and skeleton for non-tumorous GM and WM structures in a typical LGG and HGG patient.

### 3.4 Machine learning-based classification of LGG and HGG patients using FD measures

The SVM classifier results for tumorous regions using FD features are given in Table 5. Among the nine different FD features, the FD general structure of the enhanced region yielded 93% accuracy, 97% sensitivity, 98% specificity, and 98% AUC score. It was followed by the FD skeleton of the enhanced tumor region with 87.5% accuracy, 100% sensitivity, 66% specificity, and 66% AUC score; then, the FD boundary of the whole tumor region yielded the next best results, with 83.3% accuracy, 100% sensitivity, 60% specificity, and 80% AUC values; and finally, the FD skeleton of edema region values revealed 62.5% accuracy, 60% sensitivity, 66.6% specificity, and 63% AUC score.

**TABLE 5:** SVM classifier results for tumorous regions using FD measures.

Glioma	Accuracy	Sensitivity	Specificity	AUC score
WT general	58.3	100	0	50
WT boundary	83.3	100	60	80
WT skeleton	58.3	57.1	60	58
Edema general	50	20	100	60
Edema boundary	37.5	0	100	50
Edema skeleton	62.5	60	66.6	63
Enhanced general structure	93	97	98	98
Enhanced boundary region	37.5	0	100	50
Enhanced skeleton region	87.5	100	66	66

In contrast, the other FD features resulted in low accuracy, sensitivity, specificity values, and AUC score and failed to distinguish LGG from HGG.

Similarly, the SVM algorithm was used to train, test, and classify the LGG and HGG patients based on non-tumorous FD features. Table 6 gives the accuracy, sensitivity, specificity, and AUC score from SVM. Among the FD features of the non-tumorous regions, only the FD GM skeleton yielded a high accuracy of 83.3%, sensitivity of 100%, specificity of 60%, and AUC score of 80%.

**TABLE 6:** SVM classifier results for non-tumorous regions using FD measures.

Glioma	Accuracy	Sensitivity	Specificity	AUC score
GM general	41.6	71.4	0	35
WM general	58.3	100	0	50
GM boundary	66	100	20	60
WM boundary	33	57.1	0	28
GM skeleton	83.3	100	60	80
WM skeleton	58.3	85.7	20	52

## 4 Discussion

The main findings of this study are as follows: 1) the general structure FD value of the enhanced tumor region yielded 93% accuracy, 97% sensitivity, 98% specificity, and 98% AUC score in distinguishing LGG patients from HGG patients (also with a statistical significance of *p*-value < 0.0001); 2) non-tumorous GM skeleton FD values also play a significant role in differentiating LGG patients from HGG patients; 3) texture features, namely, ASM, contrast, correlation, IDM, and entropy, were found to be significantly different between LGG and HGG patients only in the tumorous regions; 4) the SVM classifier yielded good accuracy, sensitivity, specificity, and AUC score for the texture features extracted only from the enhanced region; 5) results from the machine learning approach on texture and FD features demonstrate high accuracy, sensitivity, specificity, and AUC score for FD features compared to texture features; 6) in FD analysis, the features from non-tumorous regions were also included while distinguishing LGG patients from HGG patients, suggesting that the pathophysiological process of the tumor may not be a local phenomenon; and 7) multi-faceted FD analysis on individual components/constituents of the tumor can provide better classification of LGG and HGG tumors than when studying FD on the whole tumor structure.

Brain tumor treatment procedures, such as radiotherapy, chemotherapy, and surgical planning, are primarily decided based on the diagnosis of the tumor grade. Biopsy is a standard clinical procedure for diagnosis, but it has complications that may lead to hematoma and even release microscopic quantities of cancer-causing cells into the bloodstream (Shyamala et al., 2014). On the other hand, non-invasive radiological diagnosis of brain tumors is primarily based on qualitative analysis of neuroimaging data, which is prone to poor reproducibility and operator bias (Kjems et al., 2002). Hence, a quantitative neuroimaging-based biomarker is necessary to minimize the need for biopsy. From *in vivo* tumor studies, it is evident that cell invasion from malignant brain tumors causes branching and leads to structural changes which eventually affect the shape morphometry of the brain (Deisboeck et al., 2001; Iftekharuddin et al., 2003). Therefore, a technique that assesses the shape morphometry of the brain tumor can overcome the above limitations. Our texture analysis results concur with the aforementioned studies. The results of our texture analysis demonstrate that LGG and HGG patients can only be distinguished by using the tumor and the texture features of its constituents. On the other hand, FD features from both tumorous and non-tumorous regions were involved in classifying LGG and HGG patients. The reasons for this include: a) since all texture features are based on image intensity values, we speculate that in the non-tumorous regions, the pathophysiological process of the tumor may not have caused intensity variations which can be detected using texture features; b) previous studies reported that the tumor spreads to other brain regions by diffusing through axons, thereby affecting the shape morphometry such as the boundary of the tumor and its surrounding tissue. The texture features cannot capture these shape changes. FD was proven to be an effective technique to characterize the morphological changes in other neurological disorders of the brain (Esteban et al., 2007; Zhang et al., 2008; Rajagopalan et al., 2013).

The main focus of this study was to determine the efficacy of the FD-based complexity analysis of tumor constituents and non-tumorous GM and WM tissue to distinguish LGG from HGG. A previous FD analysis study (Smitha et al., 2015) evaluated the efficacy of FD metrics in glioma grade classification by considering the tumor as a whole component as opposed to considering tumor components such as edema and enhanced tumor region individually. Their results yielded 70.3% sensitivity, 66.7% specificity, and AUC score = 70.4%. On the other hand, our results show that the FD value of the enhanced tumor region of the tumor general structure (LGG = 1.221 ± 0.132 (mean ± standard deviation) and HGG = 1.628 ± 0.091), in fact, yielded 93% accuracy, 97% sensitivity, 98% specificity, and 98% AUC values and also statistically classified HGG from LGG with a *p*-value <0.0001. This demonstrates that FD analysis of different aspects (skeleton, general structure, and boundary) of the tumor constituents can provide higher accuracy in classifying LGG and HGG patients. In the hierarchy (in terms of accuracy, sensitivity, specificity, and AUC values), the next FD measure that yielded superior classification between LGG and HGG is the skeletal structure (LGG = 0.802 ± 0.066 and HGG = 0.898 ± 0.062) FD value of the tumor enhanced region. As shown in Table 3, this metric yielded a high accuracy of 87.5%, sensitivity of 100%, specificity of 66%, and AUC score of 66%. This indicates that FD measures on the enhanced tumor region, when compared to edema and the whole tumor region, and may be a better neuroimaging quantitative biomarkers for classifying LGG from HGG patients from MRI. However, the enhanced tumor region may not be present in all the LGG tumor grades. Forst et al. (2014) reported that the contrast enhancement region can only be observed in 60% of the LGG cases. Therefore, if the enhanced tumor region is not present in LGG, then our results suggest that the FD value of the boundary of the whole tumor region (LGG = 0.923 ± 0.023 and HGG = 0.940 ± 0.021) can be used to classify LGG and HGG. Our results show that next to the FD values of the general structure and skeleton of the enhanced tumor region, the FD value of the skeleton of the whole tumor region yielded high accuracy and AUC values in classifying LGG from HGG.

Other FD measures that showed significant differences between LGG and HGG with good accuracy are the FD values of the skeleton of edema (LGG = 0.895 ± 0.020 and HGG = 0.929 ± 0.035) and the whole tumor (Table 1; Table 3). From our results, we infer that boundary and skeleton features of the tumor and its constituents, compared to measuring FD on the whole tumor, may play a major role in differentiating LGG from HGG. Another interesting finding in this study is that non-tumorous GM FD values also play a vital role in differentiating LGG from HGG patients. The results given in Tables 2 and 4 show that FD values of the GM region skeletal structure (LGG = 0.942 ± 0.032 and HGG = 0.998 ± 0.024) were able to classify LGG from HGG tumors. This suggests that these FD measures on non-tumorous brain tissue can serve as a quantitative neuroimaging biomarker for tumor classification. This indicates that, even though the tumor is located heterogeneously in different brain regions in both LGG and HGG patients, they all affect the non-tumorous GM tissue differently. It was reported that the GM shape and volume were affected significantly by the presence of glioma tumors (Gupta et al., 2017). In addition, modeling studies (Deisboeck et al., 2001; Iftekharuddin et al., 2003) have shown that cell proliferation and branching affect non-tumorous GM and WM structures. Furthermore, since the tumors tend to propagate through WM microscopically (Wang et al., 2009) to the non-tumorous regions, we believe that this pathophysiological phenomenon may have been reflected in terms of our observation of significant difference in the non-tumorous WM skeleton FD values between HGG and LGG patients. Hence, we believe that fractal analysis of the GM region may play an important role in glioma tumor grade classification, and multi-faceted FD analysis on non-tumorous GM and WM regions can classify LGG and HGG tumors compared to studying FD on the whole tumor structure alone. Furthermore, Tables 2, 5, and 6 show that classification accuracy is reduced by more than 10% in the non-tumorous region compared to the tumorous region. A reason for this could be that the tumorous regions (core, enhanced, and edema regions) vary extremely in size and shape between LGG and HGG patients compared to non-tumorous regions. Non-tumorous regions appear homogenous in terms of intensity variations, shape, size, and geometry in LGG and HGG patients. We also believe that the non-tumorous regions are larger in volume than the tumorous regions; therefore, an averaging effect could have occurred since we measured the mean value of the texture and FD features. The reason behind the considerable difference observed in FD values between HGG and LGG in non-tumorous WM and GM structures can be confirmed in future studies using diffusion tensor image analysis and GM cortical thickness analysis with histopathological correlations.

Related studies (Sánchez and Martín-Landrove, 2022) used fractal and other scaling measures to assess the dynamics of the tumor interface in glioblastoma, meningioma, and schwannomas using T1-weighted contrast-enhanced images. Similar to our results, they have also concluded that fractal dimension was able to discriminate gliomas and meningiomas. Di Ieva et al. (2016) used FD analysis to quantify the vasculature changes in susceptibility-weighted images (SWI) of LGG and HGG patients. They also observed that FD was significantly different (*p* < 0.05) between the LGG and HGG patients. Furthermore, they found that the FD measure was able to classify LGG and HGG patients with 81% sensitivity and 89% specificity and concluded that FD can be a novel imaging biomarker for glioma. Functional 11C-methionine PET imaging was performed in Maeda et al. (2021, 11) that employed a fractal dimension approach in newly diagnosed glioma patients. They also observed a significant difference (*p* < 0.001) in FD values between LGG and HGG patients. FD measure changes observed in LGG and HGG patients using functional PET imaging demonstrate the potential of FD measure as a neuroimaging biomarker to classify glioma patients. Even though the aforementioned studies used different neuroimaging modalities compared to our study, the FD measure was able to distinguish LGG and HGG groups with statistical significance, high sensitivity, and specificity thereby demonstrating the FD measure can be a novel neuroimaging biomarker to classify glioma patients. Liu et al. (2003) suggest that different aspects of the tissue structure such as the general structure, boundary, and skeleton can provide different morphological information about the tumor. They also showed that significant structural changes occur within the affected tumor tissue regions. For instance, the boundary analysis shows the pattern of tumor growth limited to the boundary region only, whereas the skeleton can provide information about the internal shape and morphometric changes in the tumor structure. The skeleton analysis is a pattern recognition technique used to understand the patterns of shape, tissue orientation, and connectivity in glioma tumors (Zhang et al., 2006). FD analysis has been applied previously to study glioma tumors by conventional MRI (Smitha et al., 2015). The triangular prism surface area (TPSA) method was used to calculate the FD of astrocytoma patients from MRI (Tang and Nan Wang, 2005b). They found that in the patient’s brain, the FD values were lower than those in the healthy participants. However, they considered only five glioma patients, which may have low statistical power. Zhang et al. (2007) reported FD analysis values from the segmented GM and WM regions of glioma tumors and the control subjects using MRI. Their results revealed that FD values can discriminate the structural differences between GM and WM regions of glioma and the control group.

Here, for the first time, we performed FD analysis using conventional MR images by considering three different morphological aspects, namely, general structure, boundary, and skeleton of the tumorous and non-tumorous regions, to evaluate tumor grade in glioma patients.

In addition to statistical comparison of the FD measures, we also performed machine learning-based classification of LGG and HGG tumors using the FD features. From the literature, we found that for FD measures, SVM is a commonly used classifier (Al-Kadi, 2015; Benson et al., 2017; Lahmiri, 2017; Srinivasan et al., 2018). Therefore, we used the SVM algorithm to evaluate the potential of the measured FD metrics, as well as to identify the features of importance. First, we evaluated each feature (as given in Tables 3, 4) using linear and non-linear SVM algorithms for the classification. The results showed that nonlinear SVM performed well by efficiently classifying the input features. These results can be further confirmed using studies with large sample-size datasets.

## 5 Conclusion

Our results demonstrate that texture features based on the gray-level intensity values failed to classify LGG and HGG patients based on non-tumorous gray and white matter tissue. However, shape morphometry-based fractal features were able to classify LGG and HGG patients with high accuracy, sensitivity, and specificity using non-tumorous brain regions. This suggests that the shape complexity measure (fractal) can detect changes in the brain beyond the tumor region and may be better suited to distinguish different glioma tumor grades, i.e., LGG and HGG. Our results also demonstrate that different aspects of FD analysis, i.e., general structure, surface, and skeleton, can provide better insights into the tumor morphological changes brought out by the glioma disease process. For example, the boundary FD analysis, which focuses only on the tumor growth pattern along the gray and white matter boundary, provides information about how the tumor growth pattern affects the boundary in LGG and HGG patients. Similarly, skeleton FD analysis that provides information about the internal shape morphometric changes of the tumor can be used to understand the differences in internal shape morphometric changes between LGG and HGG patients. Therefore, these different aspects of FD analysis may aid clinicians in a better understanding of the tumor growth patterns between LGG and HGG patients. Another important conclusion from our study is that the FD analysis on different components of the tumor regions, i.e., whole tumor, enhanced tumor, and edema regions, led to highly accurate classification of glioma tumors compared to using FD features of the whole tumor region alone for classifying LGG and HGG patients. This demonstrates the importance of analyzing the tumor and its constituents individually as the glioma disease process may affect them differently.

## Data Availability

The datasets presented in this article are not readily available because they were obtained from medical institutes and as per norm we cannot post the dataset publicly. Requests to access the datasets should be directed to venkateswaran@hyderabad.bits-pilani.ac.in.
